# Preparation of Mercurial Ointment

**Published:** 1850-01

**Authors:** Charles Brackett

**Affiliations:** Rochester, Indiana


### ARTICLE V.

*Preparation of Mercurial Ointment.*—By CHARLES BRACKETT, M.D., of Rochester, Indiana.

Noticing an article in one of your last Journals, on an improved method of making Mercurial Ointment, and knowing my way to be much superior in regard to the saving of labor, I am induced to send it you, to use as you may see proper.

To make either the Blue Mass, or Ointment, it is necessary only to apply a low degree of heat to the Confection of Roses, or Lard, before the Mercury is added; then add the metal, and triturate briskly, in a glass, or Wedgwood's Mortar, for twenty or thirty miuutes. By this time the metal is so reduced that its globules cannot be distinguished by a glass magnifying four or five times. This is the plan I have followed for the last three years; and I am convinced of its efficacy. I thought when I hit upon the plan, (which I did by accident by shaking up some mereury in a jar, containing some old ointment, which I had warmed for the purpose of getting it out,) that probably the heat dispelled the metal, but I have since carefully weighed the ingredients after being made, and have found the weight the same after admixtion, as before. I simply warm part of the lard sufficiently to make it semi-fluid, then add the metal.

